# Causal relationships between alterations in shear stress-related genes and aneurysmal subarachnoid hemorrhage

**DOI:** 10.1186/s13023-025-03784-3

**Published:** 2025-06-11

**Authors:** Xiaoxin Wu, Yuanyuan Dai, Xingyang Niu, Minghao Zhang, Jiaoxing Li, Yi Xie, Wenli Sheng, Fei Ye

**Affiliations:** 1 Department of Neurology, The First Affiliated Hospital, Sun Yat-sen University, Zhongshaner Road No.58, Guangzhou, 510080 China; 2 Guangdong Provincial Key Laboratory of Diagnosis and Treatment of Major Neurological Diseases, The First Affiliated Hospital, Sun Yat-sen University, Guangzhou, China; 3 Operating Theater, Woodlands Health, Singapore, Singapore; 4 Department of Neurosurgery, Southwest Hospital, Army Medical University, Chongqing, China; 5 Neuroscience and Behavioral Disorders Program, Duke-NUS Medical School, National University of Singapore, Singapore, Singapore

**Keywords:** Aneurysmal subarachnoid hemorrhage (aSAH), Shear stress (SS), Mendelian randomization (MR), KCNN4, UGCG

## Abstract

**Background:**

Current management of aneurysmal subarachnoid hemorrhage (aSAH) poses significant challenges, with emerging evidence implicating alterations in shear stress (SS) as critical in disease pathogenesis. However, the causal relationships and underlying molecular mechanisms remain elusive. This study aimed to elucidate the causal association of key SS-related genes with aSAH.

**Methods:**

SS-related genes were curated from the GeneCards database and transcriptomic datasets responsive to SS conditions. Expression quantitative trait loci (eQTLs) associated with these genes were identified as instrumental variables. An integrative analysis of genome-wide association study data for aSAH with eQTLs was conducted using bidirectional two-sample Mendelian randomization (MR) to identify SS-related genes causally linked to aSAH. Additionally, expression levels of identified genes were compared between ruptured and unruptured aneurysm walls. Functional assessments of these genes in vascular endothelial cells were also performed.

**Results:**

We identified 209 SS-related genes potentially implicated in aSAH pathogenesis. Using 599 eQTLs correlated with these genes as instrumental variables, MR analysis revealed that KCNN4 (OR = 0.83, 95% CI: 0.73–0.94) and UGCG (OR = 1.62, 95% CI: 1.07–2.48) were significantly associated with aSAH. Furthermore, RNA-sequencing data demonstrated elevated expression of KCNN4 and UGCG in ruptured intracranial aneurysms compared to unruptured ones. Functional experiments using siRNA-mediated knockdown in HUVECs showed that *siKCNN4* increased cell proliferation and disrupted endothelial barriers, while *siUGCG* enhanced tube formation ability but reduced migration.

**Conclusions:**

Our findings suggest a causal relationship between alterations in SS-related genes and aSAH. Specifically, KCNN4 and UGCG emerge as potential biomarkers critical in the disease progression of aSAH. These insights contribute to a better understanding of the molecular basis of aSAH and may guide future therapeutic strategies targeting SS-related pathways.

**Supplementary Information:**

The online version contains supplementary material available at 10.1186/s13023-025-03784-3.

## Background

Subarachnoid hemorrhage (SAH) is a rare yet severe cerebrovascular disorder of global public health significance, with a mortality rate ranging from 40 to 50% [1]. Aneurysmal subarachnoid hemorrhage (aSAH), predominantly caused by the rupture of intracranial aneurysms (IA) located at arterial bifurcations of the circle of Willis [2], contributes 80% of cases to SAH. aSAH has an incidence rate of 6.1 per 100,000 person-years worldwide [3, 4]. Despite advancements in medical and surgical treatments, managing aSAH remains challenging, with a notable 36% mortality rate within 30 days post-treatment and 42% of survivors experiencing long-term cognitive impairments [5, 6]. Therefore, elucidating the molecular mechanisms underlying IA rupture is essential for advancing our understanding and identifying new therapeutic targets in clinical practice.

Shear stress (SS), a frictional force exerted by blood flow on endothelial cells (EC), is particularly influential at vessel curvature, constricted artery segments, and bifurcation apexes [7]. Increasing evidence suggests that fluctuations in SS play a pivotal role in the formation and rupture of IAs [8, 9]. Recent studies have identified that elevated and sustained SS fluctuations can surpass the vessel wall’s tensile strength, thereby becoming a major risk factor for IA rupture [10]. Furthermore, studies have implicated SS-induced inflammatory response in the endothelium, activation of macrophages and pathways involving angiotensin II in IA progression, although the precise molecular mechanisms remain elusive [11, 12]. Cellular responses to SS are influenced by multiple factors, including cell type, cell phenotype, the cellular microenvironment, the intensity and type of SS, and the mechano-transduction machinery [13, 14]. Ion channels convert mechanical stimuli into electrical signals, while the extracellular matrix and integrins facilitate force transmission to the cytoskeleton, enabling intracellular signaling [15–17]. Disruptions in these components of the mechano-transduction machinery can impair cellular adaptation to SS, potentially leading to pathological outcomes. Thus, our study aims to explore SS-related gene expression changes associated with IA rupture, elucidate the causal relationship between SS and aSAH, and validate the impact of SS factors on vascular EC.

## Methods

### Data acquisition

Initially, the SS-related gene signature was identified by searching Genecards (*v5.19.0*), a searchable database of human genes [18], using the keyword “shear stress”. Subsequently, publicly available transcriptome datasets (accession numbers GSE103672 and GSE13353) were retrieved from the National Center for Biotechnology Information’s Gene Expression Omnibus repository (https://www.ncbi.nlm.nih.gov/geo/) [19]. The GSE103672 dataset comprised 20 EC samples exposed to pulsatile shear and 20 samples exposed to oscillatory shear conditions [20]. The GSE13353 dataset included 11 ruptured and 8 unruptured IA samples [21].

Genome-wide association study (GWAS) data were accessed through the OpenGWAS database (https://gwas.mrcieu.ac.uk/) [22, 23], which hosts a comprehensive collection of genetic associations from 50,044 GWAS summary datasets. Specifically, three GWAS statistics of European ancestry were extracted from FinnGen and European Bioinformatics Institute GWAS (EBI GWAS) to identify genetic variants as outcomes. The discovery set for SAH and the validation set for unruptured IA were sourced from the EBI GWAS through the MR Base catalog [24]. The SAH discovery set (ebi-a-GCST90018923) comprised 1,693 cases and 471,562 controls, while the validation set for unruptured IA (ebi-a-GCST90018816) consisted of 945 cases and 472,738 controls [25]. Additionally, GWAS data for the SAH validation cohort (finn-b-I9_SAHANEUR_EXNONE) included 2,127 cases and 216,665 controls obtained from FinnGen [26].

All correlations of expression quantitative trait loci (eQTLs) with SS-related genes were identified using data from the eQTLGen Consortium [27]. This dataset contained 16,987 genes and 31,684 cis-eQTLs identified in blood samples primarily from healthy European individuals.

This study was approved by the Independent Ethics Committee for Clinical Research at the First Affiliated Hospital of Sun Yat-sen University ([2023]382). All data analyzed in this study were publicly available, for which we had received prior approval from relevant ethics committees. All procedures performed in the study involving human participants were in accordance with the ethical standards of the institutional and/or national research committee and with the 1964 Helsinki declaration and its later amendments or comparable ethical standards.

### Identification of differentially expressed genes (DEGs) and functional enrichment analysis

Gene expression differences in RNA-seq datasets were analyzed using the “edgeR” package, which utilizes statistical methods based on the negative binomial distribution to model count variability [28]. DEGs were identified based on a false discovery rate (FDR) threshold of < 0.05. Functional enrichment analyses were performed using Metascape (*v3.5.20240101*), a web-based portal that offers comprehensive gene list annotation and analysis tools [29]. This platform integrates various analyses including gene ontology (GO) enrichment analysis, which elucidates biological processes, cellular components, and molecular functions associated with the gene list derived from genomic or transcriptomic data. Additionally, Metascape utilizes DisGeNET, a public repository of genes and variants associated with human diseases, to provide insights into disease-related functions of the identified genes.

### Candidate instrumental variable (IV) selection and Mendelian randomization (MR) analysis

SS-related eQTLs obtained from the eQTLGen Consortium were selected based on their significance threshold (*P* < 5.0 × 10^− 8^) as potential IVs. To minimize linkage disequilibrium effects, all potential IVs were clumped (R^2^ < 0.001, clumping distance = 10,000 kb). Additionally, eQTLs with an F-value less than 10 were excluded to retain significant eQTLs as IVs.

MR analysis was conducted using the “TwoSampleMR” package [22]. In the discovery set, we employed several MR analysis approaches including inverse variance weighted (IVW), Wald ratio, MR Egger, weighted median, simple mode, and weighted mode. For the validation set and reverse MR analysis, IVW based on a random effects model was utilized. Heterogeneity and horizontal pleiotropy were assessed using a threshold of 0.05. Significance (*P* < 0.05) in these tests indicated the presence of substantial heterogeneity or horizontal pleiotropy. Validation and reverse MR analyses were performed to strengthen the primary causal inferences derived from the MR analysis.

### Cell culture

Human umbilical vein endothelial cells (HUVECs) (*ScienCell*,* 8000*,* US*) were obtained from ScienCell Research Laboratories (*Carlsbad*,* CA*,* US*) and cultured in complete endothelial cell medium (ECM) (*ScienCell*,* 1001*,* US*) supplemented with 5% fetal bovine serum (FBS), 1% penicillin/streptomycin, and 1% endothelial cell growth supplement. HUVECs from passages 5 to 9 were used for all experiments.

### siRNA transfection

For siRNA-mediated silencing, HUVECs were seeded in six-well plates and cultured in 2 mL of complete ECM. When cells reached 70–80% confluence, the medium was replaced with 2 mL of serum-free ECM. Transfection was performed using 5 µL of target-specific siRNA (human KCNN4 siRNA: 5’-GAACUGGCAUUGGACUCAUGGUGCU-3’; human UGCG siRNA: 5’-GCCAGGAUAUGAAGUUGCAAAGUAU-3’) and 5 µL of Lipofectamine 3000 transfection reagent (*Invitrogen*,* L3000015*,* US*) in 500 µL of Opti-MEM (*Gibco*,* 31985070*,* US*). After 8 h, serum-free ECM was replaced with complete ECM, and quantitative analyses were conducted 72 h post-transfection.

### Immunofluorescence staining

Cell proliferation was assessed using the BeyoClick™ EdU (5-ethynyl-2′-deoxyuridine) -488 Cell Proliferation Kit (*Beyotime*,* C0071S*,* CHN*). HUVECs cultured in six-well plates were incubated with EdU (10 mM) diluted 1:1000 in ECM for 4 h. Cells were fixed, permeabilized, and EdU was labeled with Azide 488. Nuclei were counterstained with Hoechst 33,342. Imaging was performed using inverted fluorescence microscopy, and ImageJ software was utilized to calculate the ratio of EdU-positive to Hoechst-positive cells.

To visualize tight junctions, HUVECs were fixed with 4% paraformaldehyde, permeabilized with 0.2% Triton-X 100, blocked with immunostaining blocking buffer, and incubated with ZO-1 primary antibodies (*1:200*,* Thermo Fisher*,* 339100*,* US*) overnight at 4 °C. After incubation with Alexa Fluor 488-conjugated secondary antibodies (*1:500*,* CST*,* 4408*,* US*), cells were mounted with DAPI mounting medium and imaged using inverted fluorescence microscopy (*Olympus*,* IX83*,* JPN*).

### Cell migration assay

Cell migration assays were performed using two-well silicone inserts (*ibidi*,* 81176*,* GER*) to create a defined 500-µm cell-free gap. Upon reaching 100% confluence, the silicone inserts were carefully removed, leaving a 500-µm gap between the two populations of cells. HUVECs were then washed with 1× PBS and placed in serum-free ECM to promote migration. Images were captured immediately after insert removal and then every 12 h for a total of 36 h, using an inverted microscope at 10× magnification. The cell-free zone was quantified using the Wound Healing Tool in ImageJ software.

### Tube formation assay

Fifteen-well angiogenesis µ-Plates (*ibidi*,* 81506*,* GER*) were coated with diluted Matrigel (*Corning*,* 356255*,* US*). HUVECs (1 × 10^5^ cells/mL) were seeded onto the Matrigel and incubated at 37 °C for 12 h. Tube formation was visualized using an inverted microscope, and images were analyzed with Angiogenesis Analyzer in ImageJ software.

### RNA extraction and qRT-PCR analysis

Total RNA was extracted using the SteadyPure Universal RNA Extraction Kit (*AG*,* AG21017*,* CHN*) and reverse transcribed into cDNA using ABScript Neo RT Master Mix for qPCR with gDNA Remover (*ABclonal*,* RK20433*,* CHN*). Quantitative real-time PCR (qRT-PCR) was performed on a QuantStudio 5 Real-Time PCR System (*Thermo Fisher*,* US*) using SYBR Green detection (*ABclonal*,* RK21219*,* CHN*). Gene expression levels were normalized to β-ACTIN. Primers used are listed in Supplementary Table 1. Each experiment was conducted with at least three biological replicates.

### Statistical analysis

Statistical significance was defined as a *P*-value < 0.05, based on 95% confidence intervals (95% CI) that did not include one. Data analysis was performed using R program (*version 4.2.2*) or GraphPad Prism software (*version 8.0.1*).

## Results

### Identification of key SS-related genes

**Fig. 1:**
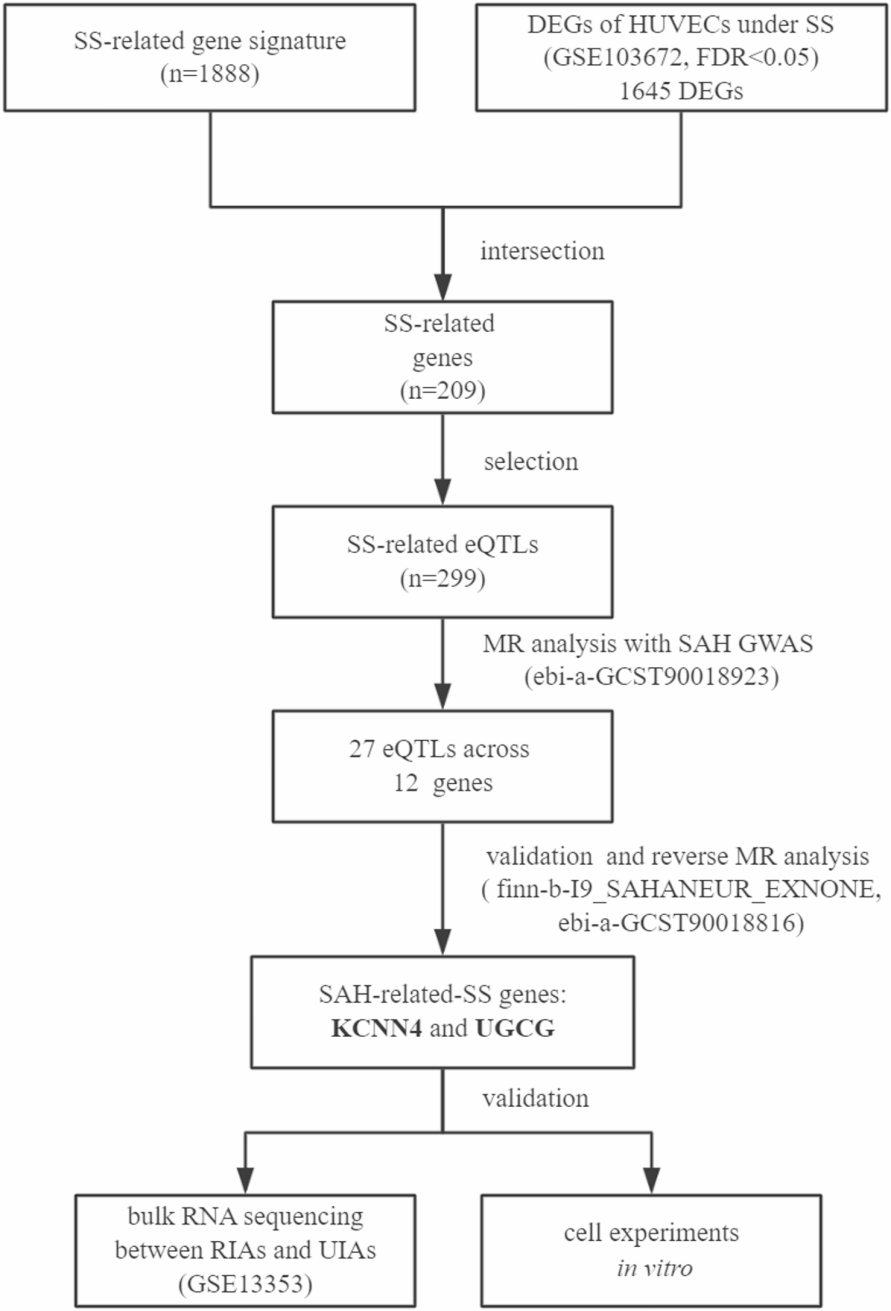
Study flowchart depicting the analysis steps to identify shear stress (SS) genes associated with aneurysmal subarachnoid hemorrhage (aSAH). 1888 SS-related gene signatures were extracted from the GeneCards database. Subsequently, a transcriptome dataset from Human Umbilical Vein Endothelial Cells (HUVECs) exposed to varying SS conditions was obtained from the Gene Expression Omnibus (GEO) database, revealing 1645 differentially expressed genes (DEGs) linked to SS. Integration of these datasets led to the identification of 209 SS-related genes. From these, 299 expression Quantitative Trait Loci (eQTLs) associated with SS-related genes were selected from the eQTLGen Consortium. Two-sample Mendelian randomization (MR) was then performed to assess the causal effect of SS-related eQTLs on aSAH. Subsequently, 27 eQTLs across 12 genes were identified and validated in a replication sample of aSAH and an unruptured intracranial aneurysm (IA) sample. Reverse MR was conducted using the aSAH dataset as exposure and the identified eQTLs as outcome variables. Finally, KCNN4 and UGCG were identified and validated through in vivo transcriptomics and in vitro cell experiments

**Fig. 2:**
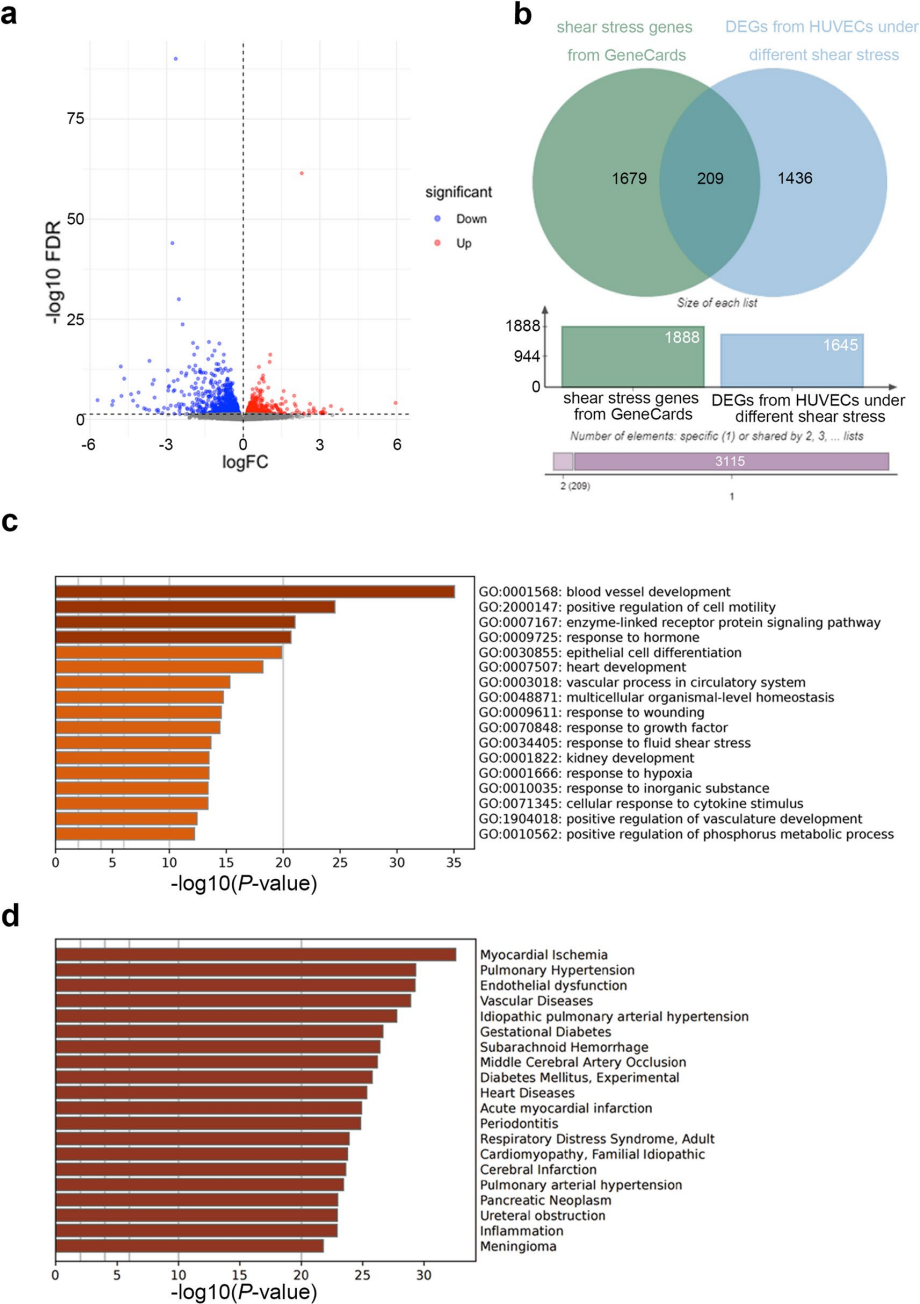
Identification of SS-related genes. (**a**) Transcriptome sequencing of HUVECs under varying SS conditions identified 1645 DEGs (FDR < 0.05). The dashed line indicates the significance threshold at FDR = 0.05. (**b**) Integration of 1888 SS genes from GeneCards with the 1645 DEGs resulted in the identification of 209 significantly SS-related genes. (**c**) Bar graph showing Gene Ontology (GO) term enrichment across the 209 SS-related genes, color-coded by *P*-values. (**d**) Enrichment analysis of the annotation of the 209 SS-related genes in the Disease and gene associations (DisGeNET) database, color-coded by *P*-values

The study flow chart is illustrated in Fig. 1. In our investigation into SS-related genes in vascular EC, we conducted a comparative analysis of transcriptomic profiles of HUVEC under varying SS conditions. This analysis revealed 1645 DEGs significantly linked to SS, as illustrated in Fig. 2(a) and detailed in Supplementary Table 2. Upon cross-referencing these findings with the established 1888 SS-related gene signature from the GeneCards database (Supplementary Table 3), we identified a total of 209 significant SS-related genes for this study, as depicted in Fig. 2(b) and summarized in Supplementary Table 4. Subsequent functional enrichment analysis of these significant SS-related genes using Metascape, a web-based platform, highlighted enrichment in GO terms associated with blood vessel development and response to fluid SS, underscoring their relevance to vascular biology (Fig. 2(c)). Additionally, examination of their disease associations through DisGeNET database, implicated these SS-related genes in potential roles within aSAH pathogenesis (Fig. 2(d)).

### MR analysis indicated a causal relationship of SS-related genes with aSAH

**Fig. 3:**
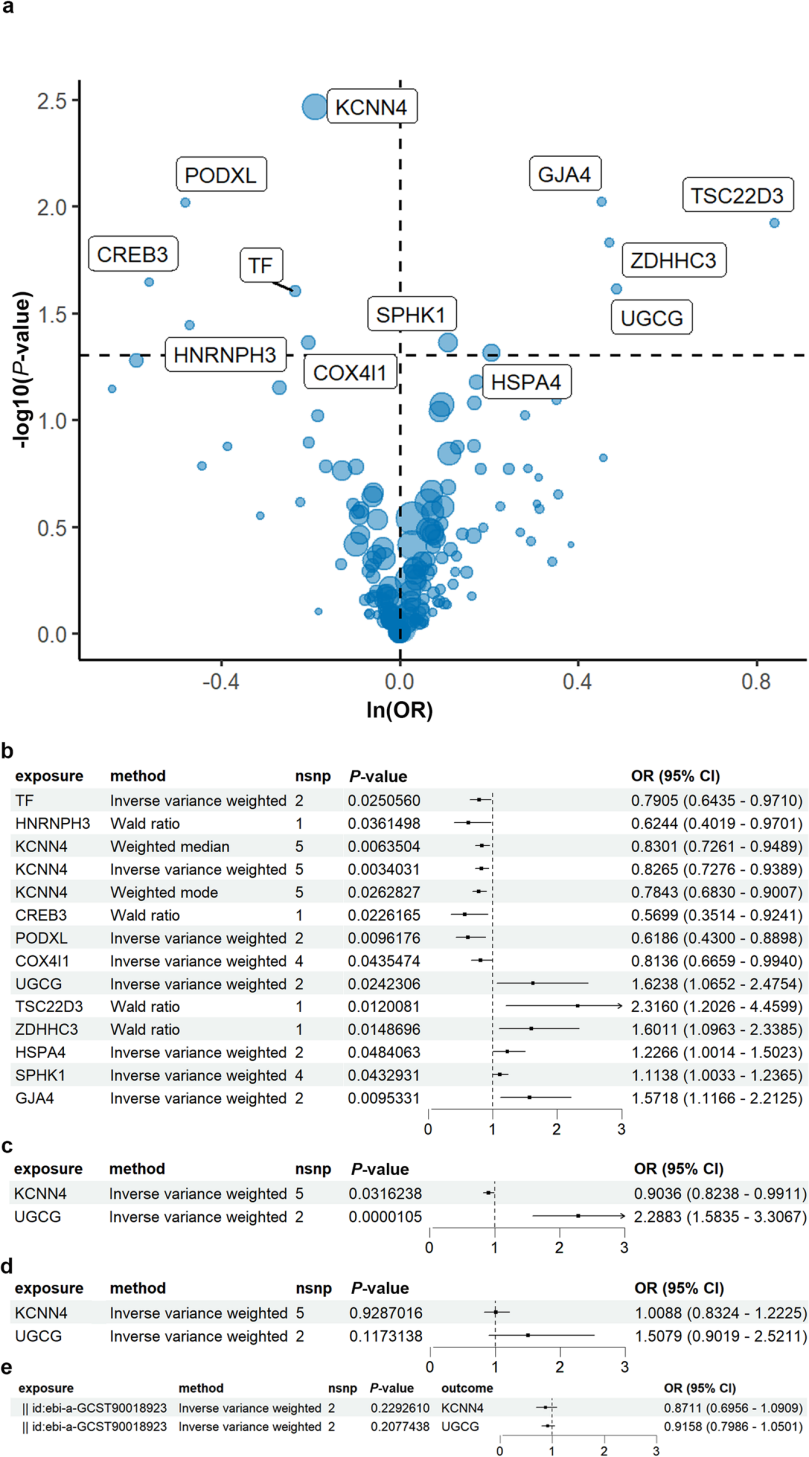
Two-sample MR analysis reveals causal SS-related genes in aSAH. (**a**) Two-sample MR analysis identifies 12 SS-related genes significantly associated with aSAH phenotype. The dashed line represents the significance threshold (*P* = 0.05) and odds ratio (OR = 1). (**b**) Forest plot illustrating MR results for the 12 SS-related genes and 27 eQTLs in the discovery set. (**c**) Forest plot showing MR results for KCNN4 and UGCG, identified as significant genes in both the discovery and validation sets. (**d**) Forest plot demonstrating the absence of causal relationships between KCNN4 and UGCG and the unruptured IA phenotype. (**e**) Forest plot displaying the results of reverse MR analysis, indicating no reverse causal relationships between KCNN4 and UGCG and the discovery set. Six MR approaches were employed, including Inverse variance weighted (IVW), Wald ratio, MR Egger, Weighted median, Simple mode, and Weighted mode, with IVW as the primary method. The number of SNPs from the outcome genome-wide association study (GWAS) is indicated in the “nsnp” column. Effect sizes are represented by black squares, with 95% confidence intervals (CI) shown as dashed lines

To investigate the association between the expression levels of SS-related genes and aSAH, we utilized the eQTL as an IV in MR analysis. In this study, we explored 599 eQTLs derived from peripheral blood, positively correlated with SS-related genes sourced from the eQTLGen Consortium. Following stringent criteria for selection, 299 eQTLs exhibited significant differences in our analysis (Supplementary Table 5). Subsequently, we applied bidirectional two-sample MR analysis to ascertain the causal relationship between these eQTLs as exposures and aSAH as the outcome. Through this analysis, we identified 27 significant eQTLs associated with 12 SS-related genes influencing the SAH phenotype (Fig. 3(a), Supplementary Table 6). Importantly, no significant heterogeneity or horizontal pleiotropy was detected among these genes. Further validation using the IVW method highlighted 7 SNPs within SS-related genes (KCNN4 and UGCG) significantly associated with SAH, with no apparent relationship observed with unruptured IAs, as depicted in Fig. 3(b), Fig. 3(c), Fig. 3(d) and detailed in Supplementary Table 6, Supplementary Table 7. Consequently, reverse MR analyses were conducted to confirm that SAH is not causally linked to SNPs from KCNN4 or UGCG (Fig. 3(e), Supplementary Table 7). In summary, these findings suggest a causal relationship between changes in SS-related genes and aSAH.

### Validation of KCNN4 and UGCG impact on aSAH *in vivo* and *in vitro*

**Fig. 4:**
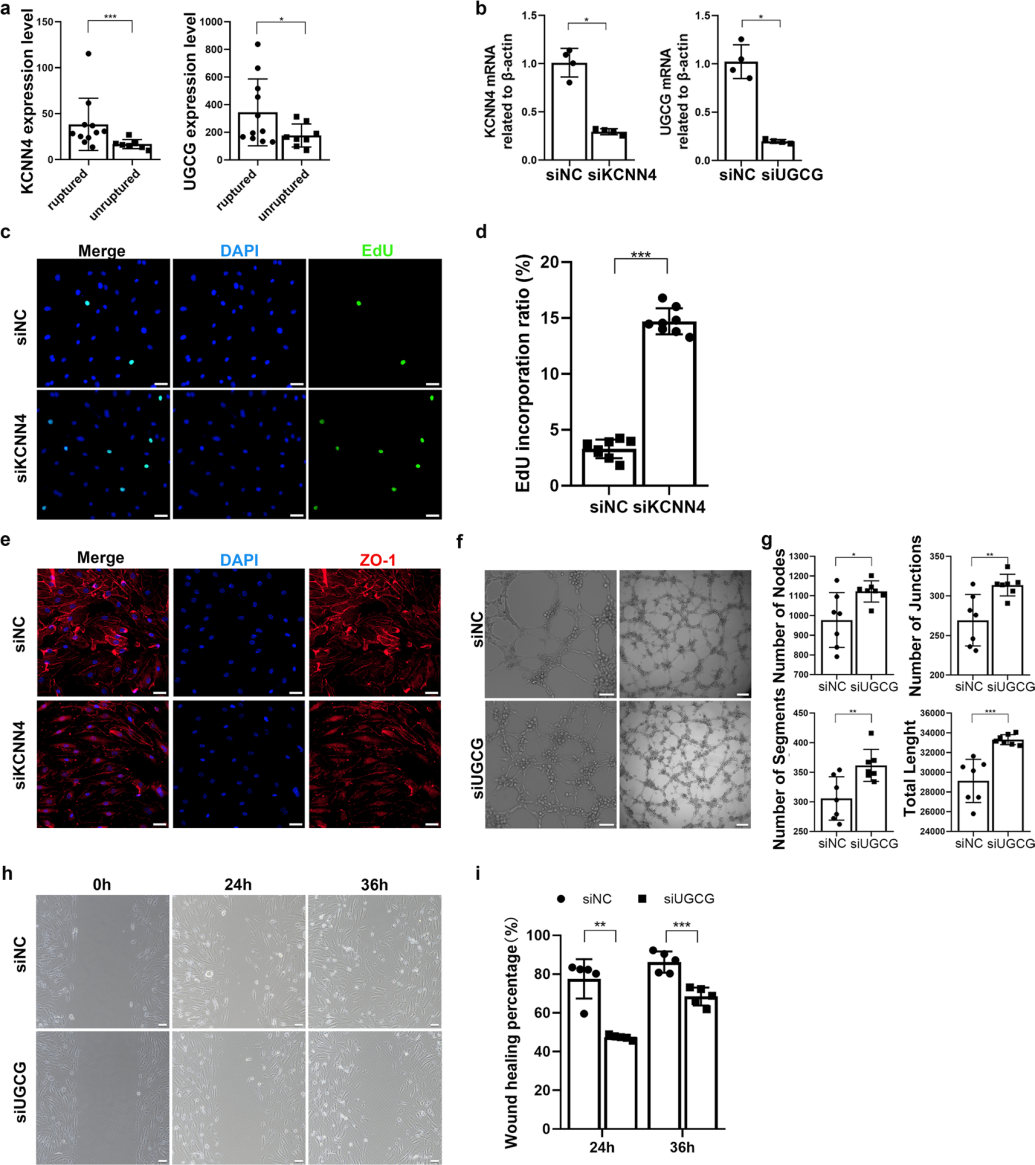
Validation of KCNN4 and UGCG impact on aSAH *in vivo* and *in vitro*. (**a**) Bar graphs showing the expression levels of KCNN4 (left, log_2_|Fold Change| = 1.18, *P* < 0.001) and UGCG (right, log_2_|Fold Change| = 0.95, *P* = 0.018) between ruptured and unruptured IAs. (**b**) qRT-PCR analysis confirming knockdown of mRNA expression levels in HUVECs using siRNA sequences (*siNC vs. siKCNN4* = 1.00 ± 0.15 vs. 0.29 ± 0.03, *P* = 0.029; *siNC vs. siUGCG* = 1.02 ± 0.17 vs. 0.19 ± 0.02, *P* = 0.029). (**c**) EdU assay measuring proliferation in *siNC*- and *siKCNN4*-treated HUVECs. Proliferative cell nuclei are stained green (EdU assay) and all nuclei blue (Hoechst). *siKCNN4*-treated HUVECs show significantly higher proliferation compared to *siNC*-treated cells (*siNC vs. siKCNN4* = 14.72 ± 1.17 vs. 3.29 ± 0.84, *P* < 0.001). Scale bar = 50 μm. (**d**) Bar plot showing EdU incorporation ratio in *siNC*- and *siKCNN4*-treated HUVECs. (**e**) Immunofluorescence staining of ZO-1 (green) and DAPI (blue) indicating disruption of tight junction proteins in *siKCNN4*-treated HUVECs. Scale bar = 20 μm. (**f**) Tube formation assay measuring angiogenesis in *siNC*- and *siUGCG*-treated HUVECs. Scale bar = 100 μm (left) and 200 μm (right). (**g**) Analysis of vessel nodes, junctions, segments, and length using Angiogenesis Analyzer for ImageJ software shows significantly increased metrics in *siUGCG*-treated HUVECs (nodes: 976.70 ± 138.80 vs. 1122.00 ± 53.20, *P* = 0.034; junctions: 269.30 ± 32.40 vs. 313.60 ± 13.70, *P* = 0.006; segments: 305.70 ± 36.70 vs. 361.60 ± 27.10, *P* = 0.007; length: 29120.00 ± 2186.00 vs. 33304.00 ± 508.10, *P* < 0.001). (**h**) Wound healing assay measuring horizontal migration of *siNC*- and *siUGCG*-treated HUVECs. Scale bar = 100 μm. (**i**) *siUGCG*-treated HUVECs show significantly reduced horizontal migration compared to *siNC*-treated cells at both 24 h (77.57 ± 10.17 vs. 47.39 ± 1.10, *P* = 0.008) and 36 h (86.20 ± 5.48 vs. 68.44 ± 4.62, *P* < 0.001). Means ± SDs are shown. *P < 0.05, ** *P* < 0.01, and *** *P* < 0.001

In this study, we aimed to validate the impact of KCNN4 and UGCG on aSAH. To assess their role in vitro, we initially analyzed gene expression differences between ruptured and unruptured IAs. Our results revealed significantly elevated levels of KCNN4 and UGCG in ruptured IAs, indicating a positive association with SAH (Fig. 4(a), Supplementary Table 8). To further validate these findings in vitro, we utilized a siRNA knockdown approach targeting KCNN4 and UGCG in HUVECs (Fig. 4(b)). Compared to the *siNC* group, the *siKCNN4* group exhibited a significantly higher proliferation rate (14.72 ± 1.17 vs. 3.29 ± 0.84, *P* < 0.0001) (Fig. 4(c), Fig. 4(d)). Immunofluorescence staining of ZO-1, a tight junction marker, indicated disrupted endothelial barriers in the *siKCNN4* group, as shown in Fig. 4(e). Furthermore, a Matrigel-coated tube formation assay was employed to assess the angiogenic capacity of HUVECs treated with *siNC* and *siUGCG*, revealing a significant increase in vessel nodes, junctions, segments, and length in the *siUGCG* group (Fig. 4(f), Fig. 4(g)). Moreover, a wound healing assay demonstrated reduced horizontal migration ability in the *siUGCG* group (Fig. 4(h), Fig. 4(i)). These findings collectively suggest that both KCNN4 and UGCG, as SS-related genes, play pivotal roles in the pathogenesis of aSAH.

## Discussion

In this study, we identified 209 SS-related genes through integrated analysis of public SS gene signatures and transcriptomic datasets responsive to SS conditions, linking them to 599 eQTLs as IVs. Among these genes, KCNN4 and UGCG have been identified as pivotal with a causal relationship to aSAH based on bidirectional two-sample MR analysis. Furthermore, validation studies conducted in vivo and in vitro confirmed that KCNN4 gene expression is negatively correlated with aSAH pathogenesis, whereas UGCG expression shows a positive correlation.

Consistent with prior research, our study reaffirms that alterations in SS are a primary cause of SAH onset and progression [30, 31]. Ruptured IA exhibit highly disturbed flow, where both low and high SS can lead to IA rupture through distinct biological mechanisms [30, 32–35]. Under low SS conditions, ECs generate reactive oxygen species, upregulate surface adhesion molecules and cytokines, facilitating leukocyte transmigration into the vessel wall [36, 37]. Conversely, high SS disrupts the internal elastic lamina and endothelium, inducing smooth muscle cell phenotype modulation [38–40]. Both leukocytes and phenotype-modulated smooth muscle cells contribute to proteolytic and oxidative damage, resulting in extracellular matrix degradation and cell death [36, 40], thereby promoting IA rupture and SAH onset. Additionally, rebleeding is a significant risk factor associated with poor prognosis and is correlated with low SS [41]. However, due to the complexity of these mechanisms, there is currently no targeted therapy directly controlling SS. Blood pressure management can reduce SS but may compromise cerebral perfusion [5]. Other medical treatments primarily target downstream molecular changes in vascular impairment resulting from SS, including aspirin, atorvastatin, matrix metalloproteinase inhibitors, free radical scavengers, and Ca^2+^ channel blockers, yet these interventions have not gained widespread clinical adoption [42–44]. In summary, accumulating evidence suggests that SS is a critical risk factor for SAH, although whether SS directly mediates SAH onset remains uncertain.

In contrast to previous findings, our study specifically delineates the causal relationship between two key SS-related genes, KCNN4 and UGCG, and SAH. KCNN4 functions as an intermediate conductance Ca^2+^-activated K^+^ channel in ECs, sensing various SS conditions induced by blood flow and initiating cytoplasmic biochemical signaling events. KCNN4 is implicated in a range of vascular diseases and stimulates cell proliferation across multiple cell types [45–50]. Under SS conditions, KCNN4 mediates membrane potential hyperpolarization in ECs, leading to smooth muscle relaxation and vasodilation [51, 52]. Additionally, KCNN4 can elongate ECs under SS conditions by interacting with scaffold-like structures [53], potentially enhancing resilience against SS. However, whether KCNN4’s actions are linked to SS regulation in aSAH remains unknown. Our results showed elevated KCNN4 mRNA levels in ruptured IAs compared to unruptured ones. In *siKCNN4*-treated HUVECs, we observed increased endothelial proliferation, potentially contributing to aneurysm formation, along with reduced expression of tight junction markers indicative of endothelial barrier disruption in aSAH pathogenesis. KCNN4 is a downstream effector of PIEZO channels [54], which are key mechanosensitive channels that respond to SS [15]. Our findings suggest that KCNN4 may function as a sensor, converting mechanical stimuli into electrical signals under the abnormal SS conditions characteristic of aSAH. This signaling may lead to changes in cellular proliferation and permeability through calcium influx.

UGCG is a crucial enzyme involved in glycosphingolipid (GSL) biosynthesis and is also implicated in oxidative phosphorylation and cell energy metabolism. GSLs are essential components of cell membranes that impact the mechanical properties of the membrane by forming raft-like lipid bilayers. Alterations in GSL composition can influence cellular mechanics by modulating the cytoskeletal structure [55]. Additionally, GSLs play a role in regulating cellular responses to mechanical forces via Ca^2+^ signaling [56]. Both UGCG and GSL influence endothelial function and mediate several vascular diseases. A previous study indicated that UGCG may contribute to venous malformation pathogenesis by affecting vascular EC activity [57]. Elevated GSL levels are associated with cardio cerebrovascular disorders [58, 59]. Inhibition of UGCG in HUVECs reduces SS-induced reactive oxygen species production [60], promoting vessel stability and mitigating damage, suggesting that UGCG functions adversely in vascular conditions. ECs with high GSL levels exhibit impaired SS responses and reduced cell streaming [61]. Using *siUGCG*-treated HUVECs, we observed decreased migratory ability and enhanced tube formation capacity, further supporting UGCG’s positive correlation with SAH onset. Moreover, elevated UGCG expression levels were noted in the ruptured aneurysm wall, reinforcing its involvement in SAH pathophysiology.

MR analysis offers several advantages in this study. First, the two-sample MR design allows for independent and robust causal estimates, effectively minimizing confounding factors that could influence the results. Second, the observed effects were specifically replicated in aSAH, but not in unruptured IAs, strengthening the causal inference. Furthermore, the use of reverse MR analysis enables us to examine whether the outcome variable influences the exposure, which helps address potential bidirectional relationships and reduces the risk of reverse causality.

There are several limitations to this study. First, we utilized eQTL data derived from blood samples instead of cerebrovascular tissue, as they are readily available in current databases. Although blood collection is minimally invasive, it still provides valuable genetic information. Second, one key assumption of MR is the absence of bias from unbalanced horizontal pleiotropy, which could distort causal estimates. While sensitivity analyses conducted in the study show consistent findings that argue against such bias, it cannot be entirely ruled out. Third, the GWAS datasets employed in the analysis are predominantly derived from European populations, which may limit the generalizability of the results to other ethnic groups. In addition, we focused solely on loss-of-function analysis of the two identified genes in HUVECs without conducting gene overexpression experiments. Extending these findings to individuals of other ethnicities requires further research and validation to ensure the broader applicability of our results.

## Conclusions

Our findings suggest a causal relationship between alterations in SS-related genes and aSAH. Specifically, KCNN4 and UGCG emerge as potential biomarkers critical in the disease progression of aSAH. These insights contribute to a better understanding of the molecular basis of aSAH and may guide future therapeutic strategies targeting SS-related pathways.

## Electronic supplementary material

Below is the link to the electronic supplementary material.

Supplementary Material 1

Supplementary Material 2

## Data Availability

The datasets used and/or analyzed during the current study are available from the corresponding author on reasonable request.

## Abbreviations

SS: Shear stress
aSAH: Aneurysmal subarachnoid hemorrhage
eQTL: Expression quantitative trait loci
IV: Instrumental variables
GWAS: Genome-wide association study
MR: Mendelian randomization
SAH: Subarachnoid hemorrhage
IA: Intracranial aneurysm
EC: Endothelial cell
DEGs: Differentially expressed genes
FDR: False discovery rate
GO: Gene ontology
IVW: Inverse variance weighted
HUVEC: Human umbilical vein endothelial cell
ECM: Endothelial cell medium
FBS: Fetal bovine serum
GSL: Glycosphingolipids
