# Supplementary material for: Causal relationships between alterations in shear stress-related genes and aneurysmal subarachnoid hemorrhage
Source: Orphanet J Rare Dis. 2025 Jun 11;20:296. doi: 10.1186/s13023-025-03784-3 (PMC12153161; doi:10.1186/s13023-025-03784-3)
Supplement: Supplementary file 1 — Supplementary Material 1 [file 13023_2025_3784_MOESM1_ESM.docx]

**Additional File**

**Causal relationships between alterations in shear stress-related genes and aneurysmal subarachnoid hemorrhage**

**Supplementary Table 1 RNA primers used in this study**

**Supplementary Table 2 1645 DEGs from transcriptomic profiles of HUVEC under varying shear stress conditions**

FC, fold change; CPM, Counts Per Million; LR, Likelihood Ratio; FDR, False Discovery Rate

**Supplementary Table 3 1888 shear stress-related gene signature from the GeneCards database**

**Supplementary Table 4 209 significant shear stress-related genes**

**Supplementary Table 5 Exposure data of 299 eQTLs**

eQTL, Expression quantitative trait loci; SNP, Single Nucleotide Polymorphism

**Supplementary Table 6 27 significant eQTLs associated with 12 shear stress-related genes influencing the SAH**

eQTL, Expression quantitative trait loci; SAH, subarachnoid hemorrhage

**Supplementary Table 7 MR results of validation**

Nsnp, Number of SNPs; b, Regression Coefficient; se, Standard Error; p value, *P*-value; pve, Proportion of Variance Explained; lo_ci, Lower Confidence Interval; up_ci, Upper Confidence Interval; or, Odds Ratio; or_lci95, 95% Confidence Interval Lower Bound for Odds Ratio; or_uci95, 95% Confidence Interval Upper Bound for Odds Ratio

**Supplementary Table 8 Expression of KCNN4 and UGCG from transcriptomic profiles of ruptured and unruptured intracranial aneurysms**
